# Monthly Summary

**Published:** 1886-01

**Authors:** 


### Monthly Summary.

Disgust for Decayed Teeth.—We recall the case of a gentleman of ability, and an earnest Christian worker, who began a worthy enterprise of some magnitude, that would be advanced by the recognition and encouragement of prominent men in the same line of effort, and he presented his plans to a man of national reputation for his endorsement. and the gentleman refused to have anything to do with it. In speaking of the matter to a friend afterward, he said, in explanation of his refusal, "What man can accomplish any good who has a mouth in such a condition, sending forth pollution at every breath?"

First impressions are very forcible, and this man will have none but a poor opinion of the good man who sought his assistance. With the work he was in sympathy, but with the medium he had disgust.

Some people are too lazy, and others think they are too busy to properly care for their teeth by brushing and cleansing them regularly; but if people could only see the condition of their mouths and teeth as easily as they can see the condition of their hands, there would be less carelessness in this respect. As a result, there would be less toothless men and women, or what is worse than toothless, people with their mouths filled with decayed and broken teeth, that stare out at the beholder like the skeletons of departed friends, standing in ghastly array, in the cavern that has become their grave.

The only rule that will apply always and everywhere for the preservation of the teeth in their natural condition, is *scrupulous cleanliness*, and even this will not always accomplish that result, but if begun in childhood and continued unremittingly there will be very little demand for the services of the dentist in extracting aching teeth. However, the teeth will decay to some extent and it becomes necessary to preserve them by artificial means, that is by filling, and every person, from the age of six years upward, should visit a competent dentist once or twice a year and have the teeth examined thoroughly, and, if any decay shows itself, have it filled at once. It is well, in this matter, to have a family dentist, just as you have a family physician, and always consult him in matters pertaining to the teeth or mouth.—*Health and Home.*

---

ZINC CHLORIDE AS A DISINFECTANT.—Dr. George H. Rohe has a note in the *Medical News* on the value of chloride of zinc for disinfection. Koch pointed out the fact that this salt is a very inefficient germicide, anthrax spores developing freely after exposure to a five per cent. solution.

Mr. A. N. Blythe says that a one per cent solution seems to stimulate the development of anthrax spores, and that it requires a twenty-five per cent. solution to arrest their growth.

On the other hand Dr, Sternberg found a two per cent. solution destructive of the micrococcus of gonorrhœal pus, while a one-half per cent. solution arrested the development of the septic micrococcus. Moreover a five per cent solution destroyed the organisms in broken-down beef-tea. As the latter organisms' were supposed to be as resistent to most germicides as those of anthrax, Sternberg, with assistance of Dr. A. C. Abbott undertook another series of experiments to clear up the discrepancy between his results and those of Koch. These experiments showed that the spores of anthrax are not killed by an exposure for two hours to a ten per cent. solution of chloride of zinc, while a five per cent solution, acting for the same time, was effective in destroying the spores of a broken-down beef-peptone solution.

The result of these experiments is to show that a five per cent. solution of zinc chloride can be relied upon to destroy micro-organisms in the abscence of spores. To destroy the vitality of anthrax spores, a twenty per cent. solution is necessary.—*N. W. Lancet.*

